# Changes in corneal endothelial cell loss in different regions in patients with cataracts and peripheral anterior synechiae after phacoemulsification combined with goniosynechialysis

**DOI:** 10.3389/fmed.2025.1615345

**Published:** 2025-11-03

**Authors:** Dan Huang, Tingting Zhu, Xiangying Luo, Ting Xi, Zhenxing Liu

**Affiliations:** ^1^Department of Ophthalmology, The Affiliated Suzhou Hospital of Nanjing Medical University, Suzhou Municipal Hospital, Suzhou, Jiangsu, China; ^2^Gusu School, Nanjing Medical University, Suzhou, Jiangsu, China

**Keywords:** corneal endothelial cell, cataracts, peripheral anterior synechiae, phacoemulsification, goniosynechialysis

## Abstract

**Purepose:**

To evaluated regional changes in regional corneal endothelial cell density and morphology during the first month after phacoemulsification with intraocular lens implantation combined with goniosynechialysis (PEI+GSL) in cataract eyes with peripheral anterior synechiae (PAS).

**Methods:**

In this single-center retrospective study, 30 patients with cataract and PAS who underwent PEI+GSL were evaluated preoperatively and 1 month postoperatively. Corneal specular microscopy (EM-4000, Tomey, Japan) was used to examine and compare the corneal endothelial cells (CECs) across seven corneal regions—central, superior, nasal-superior, nasal-inferior, inferior, temporal-inferior, and temporal-superior from preoperative and 1 month postoperative. This study analyzed the variations in corneal endothelial cell density (CD), average size (AVG), coefficient of variation (CV), and hexagonality coefficient (6A) across these regions.

**Results:**

PEI+GSL led to a reduction in corneal endothelial CD in all regions. No significant difference in endothelial cell loss was observed between the central and any individual peripheral region. However, significant differences were detected among the peripheral regions themselves. The CV also showed statistical disparities between the central and peripheral regions. No statistically significant differences were found in the AVG and 6A of corneal endothelial cells across different regions. Postoperatively, both the central and peripheral corneal endothelial CD decreased, AVG increased, 6A decreased, and CV increased.

**Conclusion:**

These findings suggest that PEI+GSL in patients with cataracts and PAS predominantly affects corneal endothelial CD and CV in the peripheral region, indicating that the surgery induces regional morphological alterations in peripheral corneal endothelial cells.

## 1 Introduction

As medical technology continues to evolve, minimally invasive and one-time combination surgeries are increasingly accepted by ophthalmologists. Numerous studies have confirmed that PEI+GSL can cause damage to the corneal endothelium, resulting in a decrease in the 6A and an increase in cell size variability (1, 2). Therefore, scholars have approached this issue from various perspectives, aiming to minimize iatrogenic corneal damage, such as the application of viscoelastic agents and the control of intraoperative ultrasound energy. When the severity of PAS is limited, laser peripheral iridotomy (LPI) alone or combined with laser iridoplasty is the preferred treatment for patients with primary angle closure/primary angle closure glaucoma (PAC/PACG), while trabeculectomy has traditionally been used to treat extensive PAS patients (3). PEI+GSL, by opening the occluded angle and deepening the anterior chamber, thus restoring trabecular function, has been proven to be effective for cataract combined with PAS/PAC/PACG, with the advantages of convenience in surgical operations and fewer complications (4). Compared with the traditional combined procedure of trabeculectomy and cataract extraction (trabeculectomy-cataract surgery), PEI+GSL has shown better outcomes in terms of postoperative anterior chamber hemorrhage, low intraocular pressure, choroidal effusion, or detachment, particularly in the shallow anterior chamber, with minimal damage (5, 6). Currently, reports regarding the alterations and patterns observed in CECs across various regions following PEI+GSL for the treatment of patients with cataracts and PAS are scarce (7). This study aimed to comprehensively evaluate the effects of PEI+GSL on CECs. This objective was achieved by analyzing changes in corneal endothelial CD, AVG, CV, and 6A across different regions following surgery. The findings of this analysis can serve as a valuable reference for ensuring safe implementation of this procedure in clinical practice.

## 2 Methods

### 2.1 Patients

This study consecutively enrolled 30 patients (23 females and 7 males) with cataracts and PAS who underwent PEI+GSL at our ophthalmology department from January to December 2022. To minimize potential confounding effects from factors such as surgical incision location, only patients who underwent right eye surgery were included. The average age was 68.8 ± 1.9 years (to minimize the influence of age on corneal endothelium). The average follow-up period was 1.07 ± 0.10 months, and the average intraocular pressure was 25.14 ± 3.80 mmHg. The inclusion criteria were as follows: Han ethnicity, aged 65–74 years, transparent cornea, preoperative best-corrected visual acuity (BCVA) ≤ 0.4, preoperative gonioscopy showing anterior chamber angle closure < 180° [according to the diagnostic and treatment guidelines for glaucoma in China (8), filtration surgery is recommended for patients with adhesions in two or more quadrants of the angle], no significant visual field damage, Emery–Little nuclear hardness grade I–III: The lens exhibited a nuclear cataract characterized by pure yellow opacification, yellow-white, or deep-yellow of the nucleus, occasionally accompanied by faint grayish cortical changes and a smooth surgical procedure. The exclusion criteria were as follows: patients with a history of ocular trauma or intraocular surgery, known eye diseases affecting the anterior segment anatomy such as ciliary or iris cysts, trauma, or use of local medications affecting the iris structure; patients with a history of uveitis; and patients on long-term use of topical or systemic corticosteroids. After applying the predefined criteria, we excluded a total of 44 cases. The specific reasons were as follows: 21 patients underwent surgery on the left eye; 8 were outside the 65–74-year age range; 2 had concomitant ocular conditions that could affect the corneal endothelium (e.g., history of uveitis or previous anterior-segment surgery); 1 exhibited preoperative corneal opacity precluding reliable specular microscopy; and 12 had incomplete clinical data or were lost to follow-up. Consequently, 30 patients were deemed eligible and included in the final analysis. All data sets were complete, with no missing values. The study was reviewed and approved by the Ethics Committee of the Suzhou Hospital Affiliated to Nanjing Medical University. After communicating relevant information, all patients voluntarily participated in the study and signed informed consent forms.

### 2.2 Surgical procedure

All the surgeries were performed by the same surgeon. Ten min before surgery, compound tropicamide eye drops were used for pupil dilation and the ocular surface was anesthetized with topical proparacaine. Transparent tunnel main incision (2.2 mm) and side-port incision was made at the corneal limbus at the 11 o‘clock and 2 o'clock position, respectively. An appropriate amount of a viscoelastic agent was injected to support the anterior chamber. Continuous circular capsulorrhexis was performed, followed by phacoemulsification and aspiration of the lens nucleus and cortex, after hydrodissection. Posterior capsule polishing was performed and an intraocular lens was implanted in the capsular bag, followed by thorough anterior capsule polishing. The viscoelastic agent behind the intraocular lens was aspirated and carbachol was injected into the anterior chamber for miosis. The viscoelastic agent used was sodium hyaluronate (Yishukang, Changzhou Pharmaceutical Research Institute Co., Ltd.), which was used to perform goniosynechialysis by separating the angles of the anterior chamber along a 360° circumference (9, 10), with a total volume of approximately (1 mL) used to maintain the anterior chamber and perform GSL. Successful synechiolysis was confirmed intraoperatively by a direct visualization with a handheld gonioscope, which revealed a deepened anterior chamber angle and the re-exposure of the trabecular meshwork. The viscoelastic agent was aspirated and the incisions were watertight. Postoperative medications included topical antibiotics for 1 week, topical nonsteroidal anti-inflammatory drugs for 3 weeks, and gradually decreasing topical corticosteroids for 1 month.

### 2.3 Collection of corneal endothelial cell morphology in different areas

**Figure 1:**
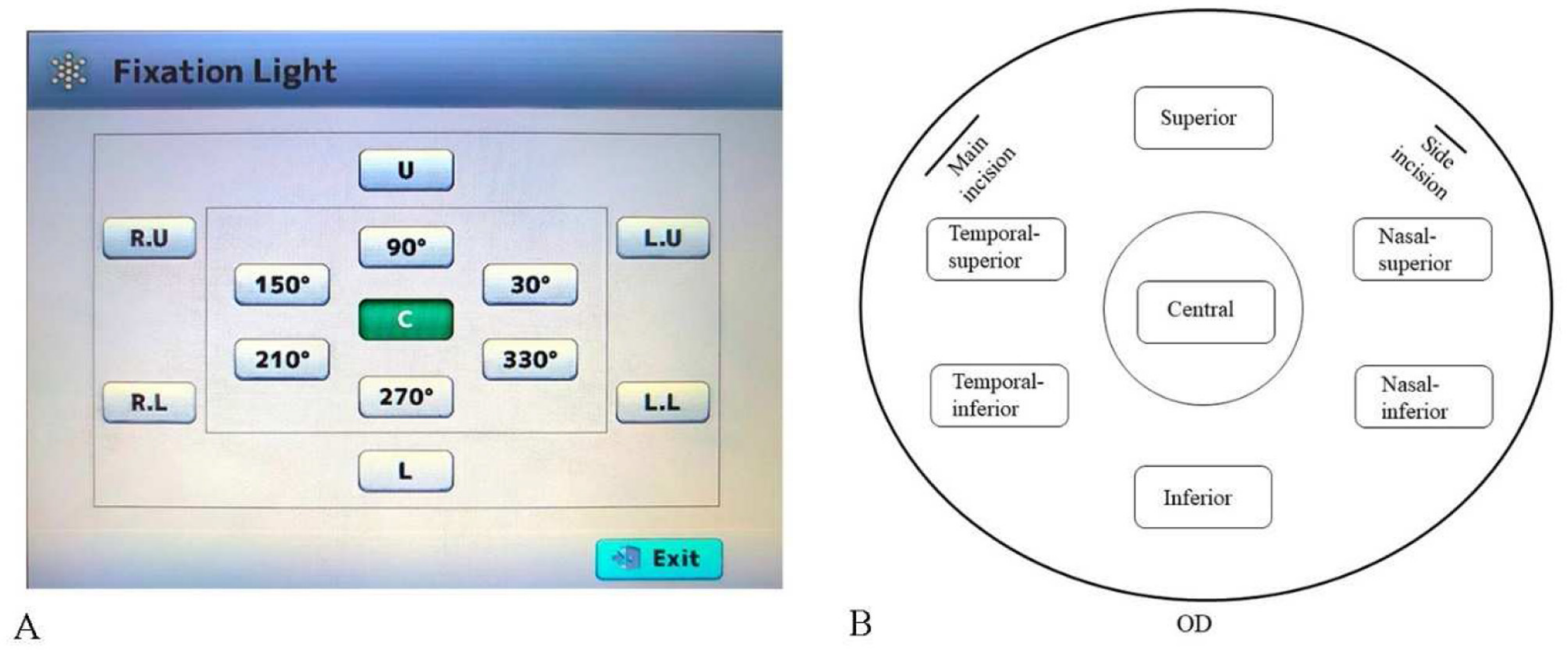
Schematic of corneal specular microscopy image acquisition. **(A)** Location and orientation of the built-in fixation light. **(B)** Diagram of the corneal regions in patients (all right eyes), indicating the locations of the main and side incisions for cataract surgery.

All operations are performed by the same examiner. To image the peripheral corneal regions, the built-in fixation light of the device was used to guide the patient's eye movement to the required positions. Automated image acquisition was performed with the corneal specular microscopy (CSM), which obtained three high-quality images from each corneal region and provided a mean value for subsequent analysis (11–14). Corneal endothelial cell morphology data, including CD, AVG, CV, and 6A, were collected using a corneal specular microscopy (TOMEY EM-4000, Japan) from the central, superior, nasal-superior, nasal-inferior, inferior, temporal-inferior, and temporal-superior regions of the operated eye's cornea preoperatively and 1 month postoperatively (Figure 1).

### 2.4 Statistical analysis

Data analysis was performed using Statistical Package for Social Sciences software (version 31.0; IBM Corp, Armonk, NY, USA). The paired *t*-test was used to compare parameters preoperative and 1 month postoperative in the same patients. Repeated-Measures analysis of variance (ANOVA) was used to compare parameter changes across the different regions from preoperative to 1 month postoperative. *Post-hoc* pairwise comparisons were performed using the Bonferroni correction. Values are presented as mean ± SD or 95% confidence intervals. Statistical significance was set at *p* < 0.05.

## 3 Results

### 3.1 Comparison of corneal endothelial cell loss

**Figure 2:**
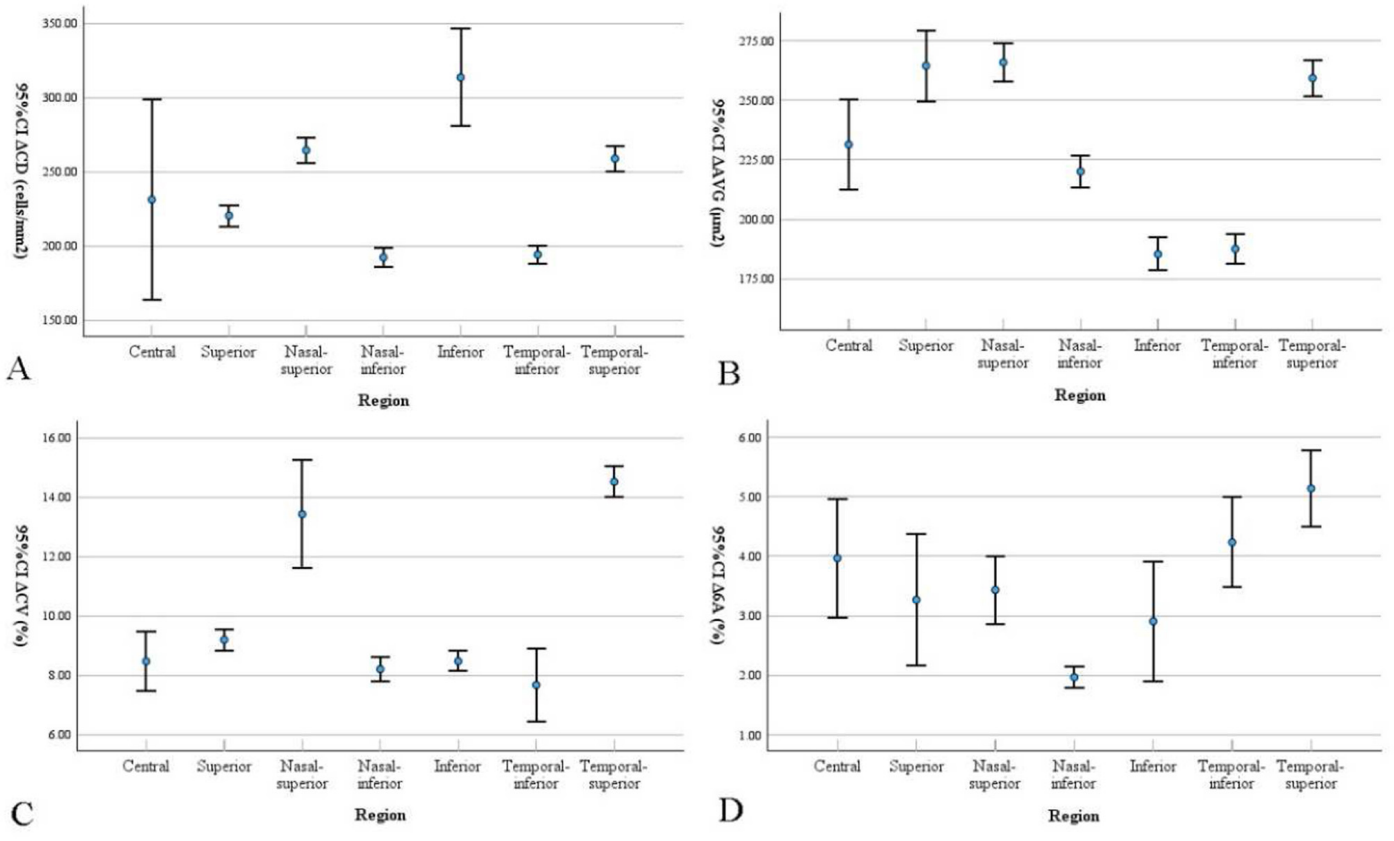
Bar graphs show corneal endothelial cell parameters changes in different regions from preoperative to 1 month postoperative. Data are presented as mean with 95% confidence intervals (CI). The Δ represents the mean difference (Preoperative - Postoperative). Differences in **(A)** cell density (CD), **(B)** average cell size (AVG), **(C)** coefficient of variation (CV), and **(D)** percentage of hexagonal cells (6A) are presented.

A total of 30 patients (23 females, 7 males) were enrolled in the study, with an average age of 68.8 ± 1.90 years. The average follow-up period was 1.07 ± 0.09 months, and the average intraocular pressure was 25.14 ± 3.80 mmHg. Patient characteristics are shown in Table 1. Statistically significant differences were found for the following parameters: BCVA, intraocular pressure (IOP), and range of PAS in the study eyes at 1 month postoperative. After surgery, the IOP and PAS range decreased, and BCVA improved, all of which were statistically significant. A comparison of corneal endothelial cell parameters in different regions from preoperative to 1month postoperative is shown in Table 2 and Figure 2. Repeated-measures analysis of variance was used to compare the differences in corneal endothelial cell parameters across different regions from preoperative to 1 month postoperative, as shown in Table 3.

**Table 1:** General information of the study participants.

**Groups**	**BCVA (Log MAR)**	**IOP (mmHg)**	**Range of PAS (degree)**
Pre-op	0.95 ± 0.50	25.14 ± 3.80	138.0 ± 28.33
1mo Post-op	0.46 ± 0.26	12.36 ± 2.31	46.67 ± 18.07
*t*	7.263	18.955	16.001
*p*	< 0.05	< 0.05	< 0.05

Data are presented as mean ± SD. Pre-op, preoperative; 1-mo Post-op, 1-month postoperative. BCVA, best correct visual acuity; IOP, intraocular pressure; PAS, peripheral anterior synechiae.

**Table 2:** Comparison of corneal endothelial cell parameters among different corneal regions at the preoperative and 1 month postoperative time points.

**Corneal regions**	**Parameters**	**CD (cells/mm^2^)**	**AVG (μm^2^)**	**CV (%)**	**6A (%)**
Central	Pre-op	2419.8 ± 247.0	398.5 ± 29.1	40.4 ± 4.4	48.0 ± 6.4
	1mo Post-op	2188.6 ± 166.2	629.9 ± 46.0	48.9 ± 4.4	44.0 ± 5.5
	Δ	231.2 ± 181.4	231.4 ± 50.6	8.5 ± 2.7	4.0 ± 2.7
	*t*-test	6.979	−25.059	−17.422	8.172
	*p* value	< 0.001	< 0.001	< 0.001	< 0.001
Superior	Pre-op	2444.6 ± 214.8	384.4 ± 38.0	42.3 ± 4.0	49.5 ± 5.8
	1mo Post-op	2224.3 ± 195.5	649.0 ± 55.9	51.5 ± 4.9	46.2 ± 6.5
	Δ	220.4 ± 19.3	264.6 ± 39.9	9.2 ± 1.0	3.3 ± 2.9
	*t*-test	62.618	−36.3	−51.9	6.071
	*p* value	< 0.001	< 0.001	< 0.001	< 0.001
Nasal-superior	Pre-op	2404.5 ± 208.1	390.9 ± 31.1	37.6 ± 2.8	49.9 ± 6.7
	1mo Post-op	2140.0 ± 185.2	656.9 ± 52.2	51.0 ± 5.2	46.5 ± 6.5
	Δ	264.5 ± 22.8	266.0 ± 21.1	13.4 ± 4.9	3.4 ± 1.5
	*t*-test	63.473	−68.983	−15.039	12.338
	*p* value	< 0.001	< 0.001	< 0.001	< 0.001
Nasal-inferior	Pre-op	2402.2 ± 207.9	399.7 ± 31.7	40.9 ± 4.0	47.8 ± 6.5
	1mo Post-op	2209.9 ± 191.2	619.8 ± 49.3	49.1 ± 5.0	45.8 ± 6.3
	Δ	192.3 ± 16.6	220.1 ± 17.5	8.2 ± 1.1	2.0 ± 0.5
	*t*-test	63.310	−68.697	−42.232	21.977
	*p* value	< 0.001	< 0.001	< 0.001	< 0.001
Inferior	Pre-op	2432.7 ± 210.5	389.2 ± 34.7	42.2 ± 4.2	50.9 ± 5.2
	1mo Post-op	2119.1 ± 209.0	574.5 ± 42.9	50.7 ± 4.9	48.0 ± 6.1
	Δ	313.64 ± 88.3	185.3 ± 18.5	8.5 ± 0.9	2.9 ± 2.7
	*t*-test	19.5	−55.0	−51.075	5.880
	*p* value	< 0.001	< 0.001	< 0.001	< 0.001
Temporal-inferior	Pre-op	2425.3 ± 209.9	392.2 ± 34.1	37.0 ± 3.3	50.8 ± 6.1
	1mo Post-op	2231.2 ± 193.1	579.8 ± 46.1	44.7 ± 4.7	46.6 ± 6.6
	Δ	194.1 ± 16.8	187.5 ± 16.5	7.7 ± 3.3	4.2 ± 2.2
	*t*-test	63.259	−62.402	−12.830	10.532
	*p* value	< 0.001	< 0.001	< 0.001	< 0.001
Temporal-superior	Pre-op	2353.6 ± 203.8	398.4 ± 31.7	38.7 ± 3.9	49.5 ± 6.5
	1mo Post-op	2094.8 ± 181.3	657.8 ± 52.3	53.3 ± 5.3	44.4 ± 6.4
	Δ	258.8 ± 22.5	259.4 ± 20.6	14.5 ± 1.4	5.1 ± 1.7
	*t*-test	63.144	−68.974	−56.3	16.4
	*p* value	< 0.001	< 0.001	< 0.001	< 0.001

Data are presented as mean ± SD. Pre-op, preoperative; 1-mo Post-op, 1-month postoperative. CD, cell density; AVG, average size; CV, coefficient of variation; 6A, hexagonality coefficient. The Δ represents the mean difference (Preoperative − Postoperative).

**Table 3:** Comparison of corneal endothelial cell parameters changes among different regions from preoperative to 1 month postoperative.

**Corneal regions**	**ΔCD (cells/mm^2^)**	**ΔAVG (μm^2^)**	**ΔCV (%)**	**Δ6A** **(%)**
Central vs. Superior	1.000	0.019^*^	1.000	1.000
vs. Nasal-superior	1.000	0.008^*^	< 0.001^*^	1.000
vs. Nasal-inferior	1.000	1.000	1.000	0.011^*^
vs. Inferior	0.391	< 0.001^*^	1.000	1.000
vs. Temporal-inferior	1.000	< 0.001^*^	1.000	1.000
vs. Temporal-superior	1.000	0.061	< 0.001^*^	0.708
Superior vs. Nasal-superior	< 0.001^*^	1.000	< 0.001^*^	1.000
vs. Nasal-inferior	< 0.001^*^	< 0.001^*^	< 0.001^*^	0.641
vs. Inferior	< 0.001^*^	< 0.001^*^	0.003^*^	1.000
vs. Temporal-inferior	< 0.001^*^	< 0.001^*^	0.104	1.000
vs. Temporal-superior	< 0.001^*^	1.000	< 0.001^*^	< 0.001^*^
Nasal-superior vs. Nasal-inferior	< 0.001^*^	< 0.001^*^	< 0.001^*^	0.002^*^
vs. Inferior	0.084	< 0.001^*^	< 0.001^*^	1.000
vs. Temporal-inferior	< 0.001^*^	< 0.001^*^	< 0.001^*^	1.000
vs. Temporal-superior	< 0.001^*^	< 0.001^*^	1.000	0.006^*^
Nasal-inferior vs. Inferior	< 0.001^*^	< 0.001^*^	1.000	1.000
vs. Temporal-inferior	< 0.001^*^	< 0.001^*^	1.000	< 0.001^*^
vs. Temporal-superior	< 0.001^*^	< 0.001^*^	< 0.001^*^	< 0.001^*^
Inferior vs. Temporal-inferior	< 0.001^*^	1.000	1.000	0.486
vs. Temporal-superior	0.033^*^	< 0.001^*^	< 0.001^*^	< 0.001^*^
Temporal-inferior vs. Temporal-superior	< 0.001^*^	< 0.001^*^	< 0.001^*^	1.000
*F* value	749.305	852.029	521.531	30.332
*p* value	< 0.001^*^	< 0.001^*^	< 0.001^*^	< 0.001^*^

^*^Indicates a statistically significant difference in *post hoc* pairwise comparisons between corneal regions (*p* < 0.05). The Δ represents the mean difference (Preoperative − Postoperative). CD, cell density; AVG, average size; CV, coefficient of variation; 6A, hexagonality coefficient.

### 3.2 Postoperative complications

Minor anterior chamber hemorrhage was observed bilaterally on the first postoperative day, without any intervention required, as it resolved spontaneously. There were no instances of severe postoperative complications such as posterior capsule rupture, malignant glaucoma, or intraocular inflammation.

## 4 Discussion

In clinical practice, corneal endothelial CD obtained through specular microscopy imaging serves as an important marker for assessing corneal endothelial function. However, corneal endothelial CD is an indirect indicator that cannot fully represent the physiological functions of corneal endothelial cells. Therefore, when corneal endothelial CD falls below critical levels and/or is under physiological stress, the potential corneal functional reserve may be insufficient to maintain adequate corneal dehydration and clarity.

To comprehensively evaluate corneal endothelial physiological function, we analyzed corneal endothelial cell morphology. Our study included participants of Han ethnicity, aged 65–74 years, with cataracts graded as Emery–Little nuclear hardness grade I–III. These criteria were chosen to reduce confounding variables and ensure homogeneity within the study population. Specifically, restricting the study to the Han ethnicity minimized the confounding influence of genetic variation on corneal endothelial cell characteristics. The age range limitation was implemented to minimize the impact of physiological aging, a known factor contributing to the progressive decline in endothelial cell density. Limiting cataract severity to grade III–IV helped control for surgical variables, as more advanced cataracts are associated with increased phacoemulsification energy, longer operative times, and potential alterations in surgical technique.

Several studies (15, 16) have found that central corneal endothelial cell loss is more severe than peripheral corneal endothelial cell loss in patients with PEI, with corneal endothelial CD stabilizing after 3 months and no effect on other endothelial stress markers up to 2 years postoperatively. Insertion of the Ex-press^®^ shunt (EXP) into the cornea or implantation of Ahmed glaucoma valve is a risk factor for rapid corneal endothelial CD loss (17, 18). During the 36-month follow-up period, there is low certainty evidence to suggest that glaucoma surgery involving long-term implants results in a greater extent of corneal endothelial CD loss than glaucoma filtration surgery without the use of implants. After surgery, the change in endothelial parameters after microinvasive glaucoma surgery (MIGS) was comparable to the ones of patients who underwent cataract surgery alone (19). The effects of the combined procedure (PEI and trabeculectomy) on corneal endothelial cell density and area did not significantly differ from those of uncomplicated PEI (2). However, another study revealed that although there was a slight decrease in corneal endothelial CD and an increase in AVG after PEI (20), the resulting cell clusters did not differ significantly in terms of area. PEI+GSL aims to minimize potential complications associated with traditional glaucoma surgery. It also has several advantages, including preservation of the conjunctiva and the ability to perform future glaucoma surgeries if needed while maintaining the anterior chamber with minimal damage to normal physiological structures. Nonetheless, manipulation in the anterior chamber, while avoiding common complications of trabeculectomy, may cause different forms of corneal endothelial cell damage. There is limited research comparing corneal endothelial cell damage in different regions after PEI+GSL, a minimally invasive procedure that can easily be combined with cataract surgery and has been shown to be effective in reducing intraocular pressure.

To reduce the influence of confounding factors, such as ultrasound parameters, cataract severity, and surgical duration, we compared the changes in corneal endothelial cells in different regions of the same group of patients before and 1 month after surgery. In our retrospective study, we observed no significant change in AVG at 1 month postoperative, but there were statistically significant differences in CV and 6A between the peripheral and central regions, indicating increased cell variability and a decreased proportion of hexagonal cells in the peripheral corneal endothelium compared to the central corneal endothelium. The increased CV in the peripheral corneal endothelial cells indicates a higher degree of cell variability, suggesting that these cells exhibit more irregular shapes and sizes than the central corneal endothelial cells. The most pronounced reduction in corneal endothelial CD was observed in the inferior region (313.64 ± 88.3 cells/mm^2^). We hypothesize that this localized loss reflects the direction of instrument movement or fluid turbulence during phacoemulsification; however, this speculation must be confirmed by future studies that incorporate systematic review of surgical video recordings. This irregularity could be the result of cellular stress or damage caused by the surgical procedure. This may also indicate a reduced functional capacity of the peripheral corneal endothelium to maintain corneal hydration and clarity.

This study's limitations pertain mainly to its sample size and follow-up duration. The small sample size may affect the statistical robustness and generalizability of our conclusions. Another limitation is the relatively short follow-up period (1 month). Although previous studies indicate that corneal endothelial cell density usually stabilizes by 3 months postoperatively (15), our objective was to precisely characterize early regional endothelial responses. Consequently, our findings reflect morphological changes at this early time-point; their long-term stability must be confirmed in future investigations incorporating 3-, 6-, and 12-month follow-up visits. Furthermore, while we delineated the specific morphological alterations in peripheral corneal endothelium, a more comprehensive model including factors such as operative time and refractive status should be explored in future research.

## 5 Conclusions

Our findings indicate that morphological changes in corneal endothelial cells vary between the peripheral and central regions. This suggests that, while the peripheral corneal endothelium faces a certain stress risk during PEI+GSL, the robust compensatory capacity of corneal endothelial cells ensures that this risk remains within acceptable limits. These findings emphasize the importance of meticulous consideration and monitoring of the peripheral corneal endothelium during surgical procedures to prevent potential regional corneal endothelial decompensation and to preserve optimal corneal function.

## Data Availability

The raw data supporting the conclusions of this article will be made available by the authors, without undue reservation.
